# Novel Approaches for Fungal Transcriptomics from Host Samples

**DOI:** 10.3389/fmicb.2015.01571

**Published:** 2016-01-19

**Authors:** Sara Amorim-Vaz, Dominique Sanglard

**Affiliations:** Institute of Microbiology, University Hospital Center, University of LausanneLausanne, Switzerland

**Keywords:** *Candida*, transcriptomics, RNA-seq, nanoString

## Abstract

*Candida albicans* adaptation to the host requires a profound reprogramming of the fungal transcriptome as compared to *in vitro* laboratory conditions. A detailed knowledge of the *C. albicans* transcriptome during the infection process is necessary in order to understand which of the fungal genes are important for host adaptation. Such genes could be thought of as potential targets for antifungal therapy. The acquisition of the *C. albicans* transcriptome is, however, technically challenging due to the low proportion of fungal RNA in host tissues. Two emerging technologies were used recently to circumvent this problem. One consists of the detection of low abundance fungal RNA using capture and reporter gene probes which is followed by emission and quantification of resulting fluorescent signals (nanoString). The other is based first on the capture of fungal RNA by short biotinylated oligonucleotide baits covering the *C. albicans* ORFome permitting fungal RNA purification. Next, the enriched fungal RNA is amplified and subjected to RNA sequencing (RNA-seq). Here we detail these two transcriptome approaches and discuss their advantages and limitations and future perspectives in microbial transcriptomics from host material.

## Introduction

Fungal pathogens of mammals are able to live and proliferate in a wide range of host body sites including skin surfaces and mucosa, but also internal organs. In order to successfully colonize or infect tissues offering such different conditions, pathogenic fungi need effective adaptation mechanisms. Adaptive processes are controlled by transcriptional programs and their understanding can provide critical clues in fungal pathogenesis (Odds, 1988; Pfaller and Diekema, 2010).

Fungal transcriptomics in the host has already been addressed in different studies in several pathogens including *Cryptococcus neoformans* and *Aspergillus fumigatus* (McDonagh et al., 2008; Chen et al., 2014). These two fungal species are important pathogens causing high mortality in immune-compromised patients (Brown et al., 2012). These studies used microarrays and RNA sequencing (RNA-seq) approaches. In *C. neoformans*, the transcriptome in the host was performed from cerebrospinal fluid (CSF) from two AIDS patients with cryptococcal meningitis prior to antifungal therapy. The RNA was extracted from fungal cells obtained after CSF centrifugation that corresponded to 10^6^–10^8^ cells. Due to this enrichment step, the RNA extraction yielded almost only fungal material which could be further processed for direct RNA-seq analysis. The authors could analyze the profile of about 97% of all *C. neoformans* genes (from a total of about 6800 genes). Some genes were identified as significantly upregulated *in vivo* as compared to *in vitro* conditions and were genes previously recognized as contributing to pathogenicity. For example, genes with known stress response functions, such as *RIM101* (a pH-dependent regulator), *ENA1* (an ATPase transporter gene) and *CFO1* (a ferroxidase) as well as several transporters were upregulated in host samples (Chen et al., 2014). In *A. fumigatus*, the transcriptome in the host was approached using experimental nasal instillation to result in pulmonary infection in mice (McDonagh et al., 2008; Bertuzzi et al., 2014). *A. fumigatus* cells were recovered and enriched from bronchoalveolar lavage (BAL) samples from which RNA was directly extracted. Microarray hybridizations were carried out after amplification of *A. fumigatus* RNA. This approach allowed the resolution of 95% of the *A. fumigatus* genes (from a total of about 9000 genes; McDonagh et al., 2008). The *in vivo* transcriptional approaches with *A. fumigatus* allowed to perceive iron limitation, alkaline stress and nutrient adaptation as important host-dependent stresses during early stage *A. fumigatus* infection. They also revealed a biased distribution of host-response genes in subtelomeric regions of chromosomes (McDonagh et al., 2008; Bertuzzi et al., 2014). In a review published by Cairns et al. (2010), *C. neoformans* and *A. fumigatus* data were compared with each other to conclude about a high degree of convergence between the *in vivo* transcriptional data from the two pathogens. Even though the *in vivo* conditions were quite different between the two experimental systems, carbon metabolism was remarkably shifted to the glyoxylate cycle in the two fungal pathogens (Cairns et al., 2010).

One of the most common fungal pathogens is *Candida albicans*, which can cause systemic infections in immunocompromised patients with mortality rates of around 50% (Odds, 1988; Pfaller and Diekema, 2010), and it is a great example of a microorganism with remarkable adaptation capabilities. Some studies have attempted to characterize the transcriptional response of *C. albicans* during the infection process. On the opposite to *C. neoformans* and *A. fumigatus* cells which can be collected from host fluids to significant numbers, *C. albicans* sampling from the host is more problematic since *C. albicans* cells in the host are associated to host tissues or embedded in organs. This motivated *in vitro* transcriptional profile experiments in which *C. albicans* growth conditions can mimic stresses encountered by the fungus within its host (see **Table 1** for details of significant studies). Alternatively, other studies have co-cultured *C. albicans* with mammalian cells or tissue cultures to obtain transcript profiles reflecting the adaptation of this fungal pathogen to different host cell types (see **Table 1**). In these conditions, the recovery and enrichment of *C. albicans* cells is not technically difficult. These data may partially reflect the real gene expression landscape of *C. albicans* in the host. It is now understood that transcriptional networks can be shaped by different profiles between *in vitro* and *in vivo* experiments, thus highlighting the value of conducting such studies directly during infection (Fanning et al., 2012; Xu et al., 2015). For example, there is only one commonly regulated transcription factor (*SFU1*, which is involved in iron homeostasis) between the *in vitro* and *in vivo* response to caspofungin, although 18 and 13 transcription factors genes are each upregulated by caspofungin *in vivo* and *in vitro*, respectively (Xu et al., 2015). Furthermore, the exploration of transcriptional data from *in vivo* conditions and their interpretation for biological relevance depends to some extent on the type of applied reference conditions (in most cases logarithmic growth *in vitro*; Cairns et al., 2010).

**Table 1:** Representative *in vitro* and *ex-vivo* transcriptomic analysis performed with *Candida Albicans.*

*In vitro* condition	*Ex vivo* condition	Sampling time points	Expression analysis	Number of regulated genes^a^	Reference
Shift to serum at 37°C		30 min, 6 h	Microarray (6580 ORF)	742	Nantel et al., 2002
Heat stress, osmotic stress, oxidative stress		10, 30, 60 min	Microarray (6580 ORF)	972	Enjalbert et al., 2003
pH shift (pH 4 vs. pH 8)		4 h	Microarray (6175 ORF)	1084	Bensen et al., 2004
Biofilm formation		24, 48, 72 h	Microarray (5907 ORF)	748–856	Garcia-Sanchez et al., 2004
Nitric oxide exposure		10, 40, 70, 120 min	Microarray (6550 ORF)	131	Hromatka et al., 2005
Tetracycline-dependent *UME6* expression		3, 10 h	Microarray (6346 ORF)	238	Carlisle and Kadosh, 2013
Low vs. high iron		5 h	Microarray (6111 ORF)	521	Chen and Noble, 2012
Spider medium		8 h	nanoString (293 ORF)	NA^b^	Finkel et al., 2012
	Human blood	10, 20, 30, 60 min	Membrane arrays (2002 ORF)	640	Fradin et al., 2003
	Human Blood fractions (PMN, MNC, Plasma)	30 min	Microarray (6039 ORF)	1518	Fradin et al., 2005
	Human neutrophils, monocytes	60, 80 min	Microarray (6550 ORF)	246	Rubin-Bejerano et al., 2003
	Murine macropahges	1, 6 h	Microarray (7600 ORF)	545	Lorenz et al., 2004
	Reconstituted human oral epithelium	1, 3, 6, 12, 24 h	Microarray (6039 ORF)	164 (upregulated)^c^	Zakikhany et al., 2007
	Reconstituted human oral epithelium	30 min	Microarray (6320 ORF)	268	Spiering et al., 2010
	Perfused pig liver	12 h	Microarray (6039 ORF)	63	Thewes et al., 2007
	Human oral epithelial cells	20, 60, 180 min	Microarray (6039 ORF)	607	Wächtler et al., 2011
	Murine dendritic cells	30, 60, 90, 120 min	RNA-seq	545	Tierney et al., 2012
	Human epithelial cells	45, 90, 180 min	Microarray (6266 ORF)	44–242	Park et al., 2009
	Human endothelial cells	45, 90, 180 min	Microarray (6266 ORF)	54–63	Park et al., 2009
	Human endothelial cells	1, 5, 5, 8 h	RNA-seq	15–31	Liu et al., 2015
	Human epithelial cell	1, 5, 5, 8 h	RNA-seq	21–63	Liu et al., 2015

*^a^Include genes that are ≥2-fold up- and down-regulated.*

*^b^NA, not available.*

*^c^Only upregulated genes were available.*

Even though it is technically challenging, several transcriptional studies have been performed on mice organs after systemic infection with *C. albicans* (Andes et al., 2005; Thewes et al., 2007), or mice feces after GI-infection (Rosenbach et al., 2010), as well as on biofilms grown on bloodstream-placed devices (Nett et al., 2009; see **Table 2** for details of significant studies). The different attempts to resolve the *C. albicans* transcriptome *in vivo* have turned out to be a great challenge for researchers since fungal RNA ratios in recovered infected organs were very low as compared to host RNA. Low fungal RNA ratios in transcript profile experiments that use microarray compromise data quality due the low signal/noise ratio. Likewise, when using RNA-seq approaches, the number of fungal reads may be too low for a comprehensive coverage of the fungal ORFome.

**Table 2:** Representative *in vivo* transcriptomic analysis performed with *C. albicans.*

Host	Route of infection	Time points	Organ/tissue/device	Expression analysis	Nucleic acids amplification	Number of regulated genes^a^	Reference
Human	NA^b^	NA	Oral cavity	Microarray (6039 ORF)	No	189 (upregulated)	Zakikhany et al., 2007
Neutropenic mice	IV infection^c^	6, 9, 15 h	Kidneys	Microarray (6737 ORF)	No	652	Andes et al., 2005
Immuno suppressed mice	OP infection^d^	1, 5 days	Tongue	nanoString (134 ORF)	No	65 (vs. Spider medium)	Fanning et al., 2012
Mice	IP infection^e^	0.5, 3, 5 h	IP space	Microarray (6039 ORF)	Yes	476	Thewes et al., 2007
Antibiotic-treated mice	GI infection^f^	3 days	GI tract (cecum)	Microarray (6333 ORF)	No	440	Rosenbach et al., 2010
Mice	Central venous catheter	12, 24 h	Catheter	Microarray (6737 ORF)	No	545 (12 h) 1034 (24 h)	Nett et al., 2009
Rabbit	IV infection	3 days	Kidneys	Microarrays (6580 ORF)	No	108	Walker et al., 2009
Zebra fish	IP infection	0.5–18 h	Whole fish	Microarray (6205 ORF)	No	120 (0.5–2 h)	Chen et al., 2013
Mice	IP infection	48 h	IP space	nanoString (145 ORF)	No	NA	Cheng et al., 2013

*^a^Include genes that are ≥2-fold up- and down-regulated.*

*^b^NA, not available.*

*^c^Intravenous infection.*

*^d^Oro-pharyngeal infection.*

*^e^Intra-peritoneal infection.*

*^f^Gastro-intestinal infection.*

Up to now, different strategies were developed to overcome this problem, including isolation of fungal cells prior to RNA extraction (Andes et al., 2005), or specific fungal RNA amplification post-RNA extraction (Thewes et al., 2007; **Table 2**). Enrichment of cells before RNA extraction exposed them to environmental changes before stopping transcription and RNA degradation, thus potentially modifying the observed transcriptional response (Andes et al., 2005). RNA amplification may be biased by non-linear amplification of fungal RNA because of the presence of large amounts of host RNA (Thewes et al., 2007). Alternative animal models have also been used such as rabbits (Walker et al., 2009) and zebrafish (Chen et al., 2013) in order to recover higher fungal biomass and to be able to perform direct transcript profiling analyses on fungal RNA.

Most of the studies mentioned so far used microarrays to measure *C. albicans* transcriptional activity, which is a method with relatively low sensitivity in quantifying the absolute expression values and in the detection of low abundance genes (Draghici et al., 2006). With more recent technologies such as RNA-seq, the detection threshold of non-aboundant transcripts has been decreased as compared to microarrays (Seqc/Maqc-III-Consortium, 2014). RNA-seq is based on established high-throughput DNA sequencing technologies that are now mainly implemented in Illumina sequencing instruments that produce high read numbers (10^6^–10^7^ per sample). RNA-seq has been used in several genome-wide *C. albicans* transcriptional studies *in vitro* (Bruno et al., 2010; Dhamgaye et al., 2012; Hnisz et al., 2012) or with *C. albicans*-infected mammalian cells with different resolutions (**Table 1**; Tierney et al., 2012; Liu et al., 2015). So far, only two studies have attempted the analysis of the *C. albicans* transcriptome by RNA-seq directly from host infections (Bruno et al., 2015; Liu et al., 2015). These reports used either infected human samples or samples from mice systemically infected with *C. albicans*. However, these studies were confronted with the low fungal transcripts proportion in the total extracted RNA. These samples had limited sequencing depth, thus resulting in the detection of a small number of highly expressed genes only.

## Novel Approaches in Fungal Transcriptome Profiling in the Host

**FIGURE 1:**
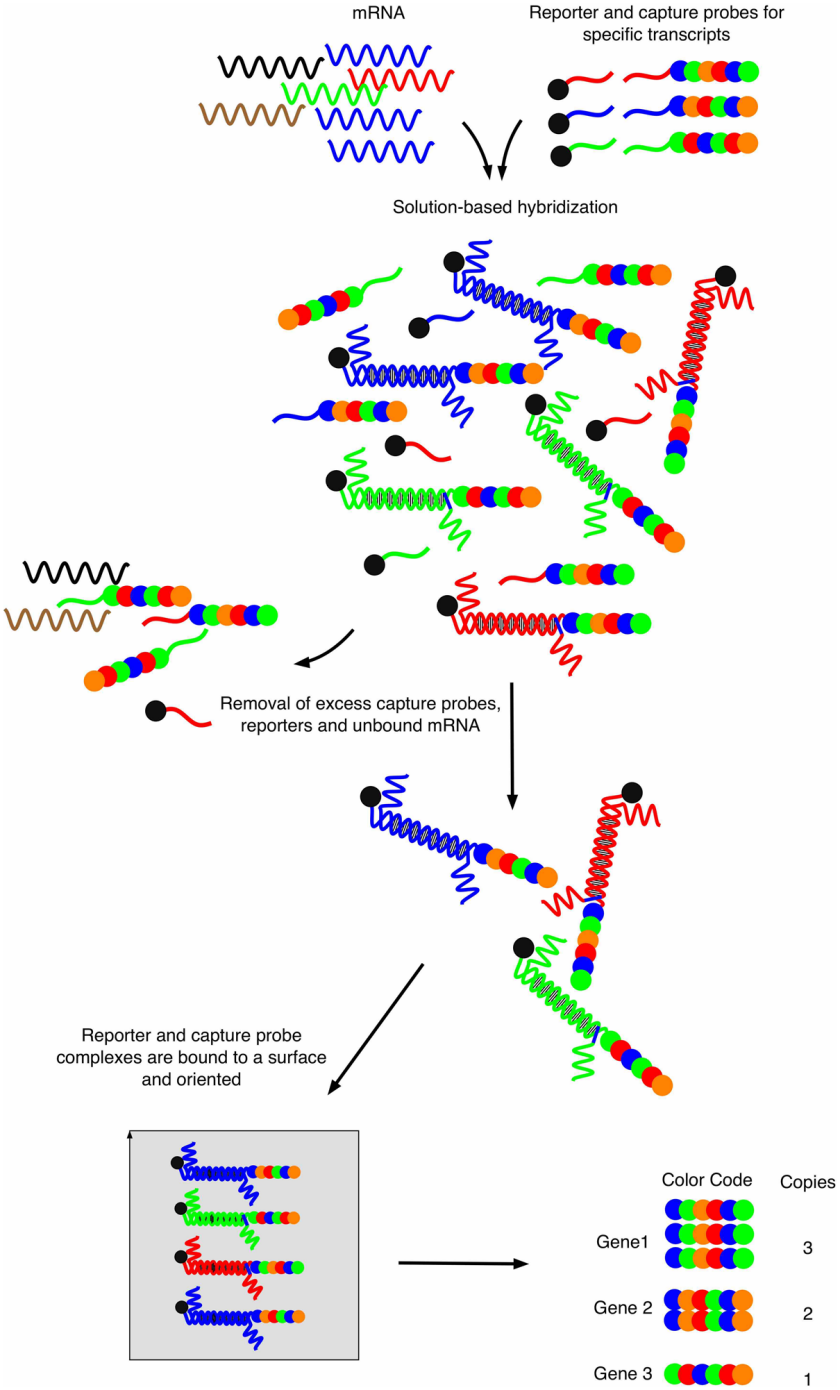
**Workflow of the nanoString strategy.** Capture probes are bound to biotin and reporter probes consist of four different fluorophores that are arranged at six different positions, thus providing a specific fluorescent signature for each probe. After mRNA hybridization and removal of excess unbound material, hybrid molecules are fixed onto a surface using electrical current which will orient all molecules. Sequential reading with a fluorescent source provides signal signatures for each specific mRNA in a quantitative manner. Adapted from Fortina and Surrey (2008).

Novel approaches to enable transcript profiling directly from host samples have emerged recently. The first technology, also called “nanoString” (Geiss et al., 2008), consists upon two key steps (**Figure 1**). Briefly, two probes are specifically designed for each target gene. One probe, called the capture-probe, is linked to biotin and helps to immobilize the molecules of interest onto a counting stand. The second probe is target-specific and is called the reporter probe. This probe is made of six fluorochromes of four different colors, defining a fluorochrome code specific to each target molecule. This color code confers the technique a very high sensitivity and enables the analysis of quantity-limited biological samples. The mRNAs of selected genes can hybridize to both their corresponding capture-probe and the reporter probes, which are both in excess in the reaction liquid. The main interest of this detection method is that it is direct and does not require linear (array) or exponential (PCR) amplifications. The method provides simultaneous digital readouts of the relative abundance of the selected mRNA species within a sample. The restricted number of sample manipulation steps together with the absence of enzymatic reaction (in most cases) allows precise and physiologically correct quantifications from starting material.

The nanoString technology was adapted to *C. albicans* and allowed the fungal transcription profiling on mice samples containing less than 0,1% of *C. albicans* RNA (Fanning et al., 2012). Other studies have used the nanoString technology but targeting a restricted number of *C. albicans* genes (248 out of a total of 6218 ORFs) from host samples originating from systemic and intra-peritoneal candidiasis in animal models (Finkel et al., 2012; Cheng et al., 2013; Xu et al., 2015). The 248 genes comprised environmentally responsive genes chosen from published genome-wide datasets. A second panel of *C. albicans* gene targets was based on the entire set of *C. albicans* transcription factors (231; Xu et al., 2015). In addition, Chung et al. (2014) reported the use of nanoString for analysis of *A. fumigatus* expression from an *in vivo* murine model of invasive pulmonary aspergillosis. Here the authors used a set of 60 different probes only (Chung et al., 2014). There is no doubt that nanoString is a powerful technology with high multiplexing capability and which overcomes the problem of low fungus/host RNA ratio. Still, this technology is provided by a single supplier, it is limited to a maximal number of target genes (800) and thus cannot yield a comprehensive transcriptional profile (Geiss et al., 2008).

**FIGURE 2:**
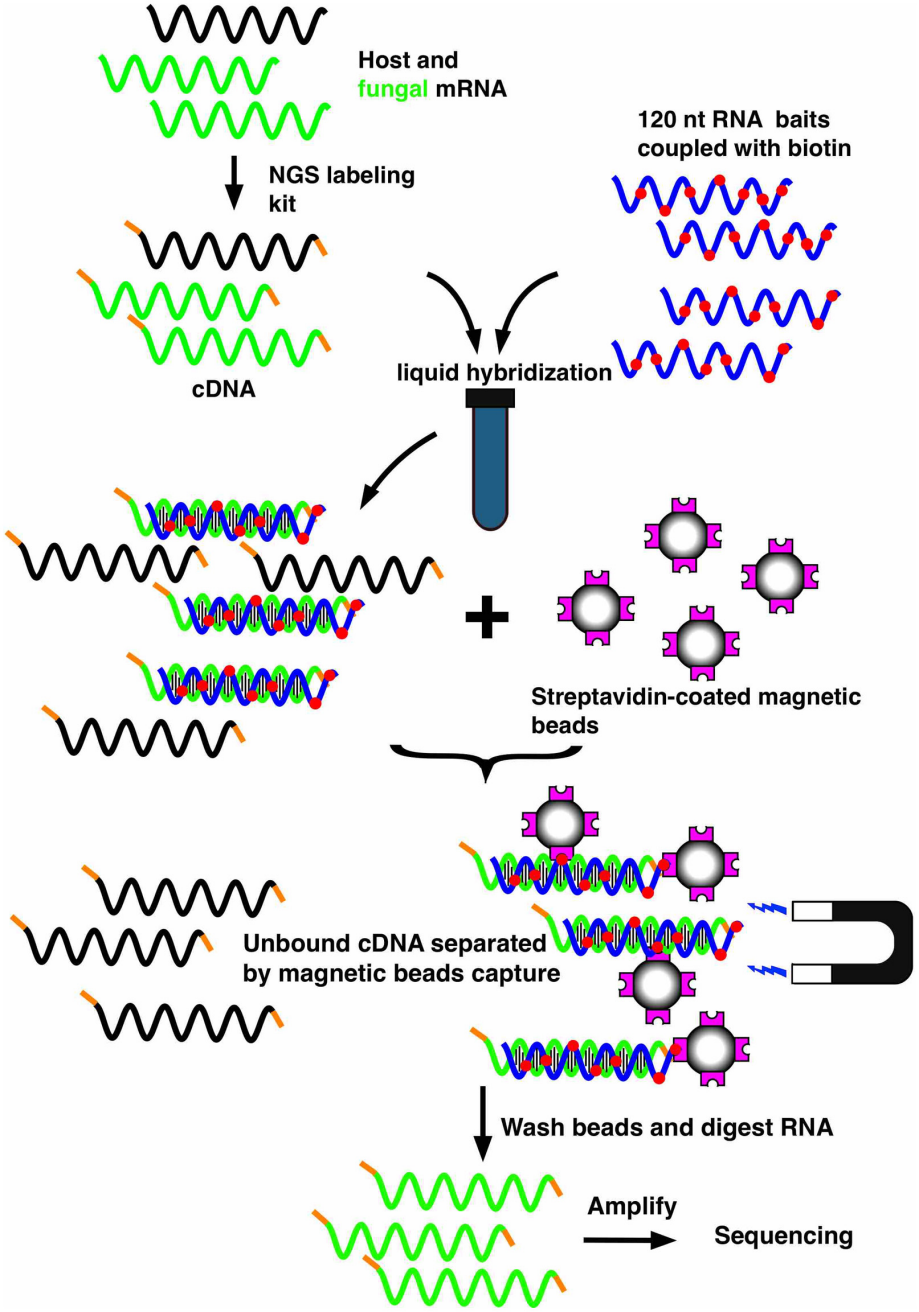
**Workflow of the mRNA enrichment strategy using the SureSelect technology.** The technology consists of capturing the mRNAs of interest out of the population of total RNA molecules transcribed from mixed genomes. A library of biotinylated oligonucleotides (corresponding to an ORFome and consisting of overlapping oligonucleotides for each gene) is hybridized in a solution with a sample (cDNA from total RNA). Biotylinated RNA-cDNA hybrids can be selectively captured with magnetic streptavidin-coated beads. After digestion of the biotinylated RNA baits, the remaining cDNA can be sequenced by high throughput sequencing approaches (RNA-seq).

Another technology that enables specific enrichment of a microbial transcriptome in a host is the bait capture method (SureSelect, Agilent), whose principle is outlined in **Figure 2**. This method was originally used to analyze the human exome (Bowne et al., 2011; Diaz-Horta et al., 2012; McDonald et al., 2012; Chilamakuri et al., 2014) and consists of capturing the sequences of interest out of the population of total RNA molecules transcribed from the whole human genome. The hybridization is carried out in a solution containing a library of biotinylated oligonucleotides corresponding to the human exome and the sample (cDNA from total RNA). The biotylinated RNA-cDNA hybrids are next selectively captured using magnetic streptavidin beads. After extensive washing steps, the remaining cDNAs can be released after digestion of the biotinylated RNA baits and are further processed for deep sequencing. We have recently adapted this technology to *C. albicans*, by designing oligonucleotides complementary to the ORFome of this fungus (Amorim-Vaz et al., 2015b). Using an online accessible platform (https://earray.chem.agilent.com/earray/), the design of biotinylated oligonucleotides can be undertaken and customized. A total of 55,342 bait probes were designed to cover 6,094 *C. albicans* ORFs. Due to cost limitations, the first 250 nucleotides of each gene were not covered in the bait design, resulting in an average of nine probes for each ORF. The use of this capture system on RNA obtained from infected host tissues resulted in enrichments in the proportion of *C. albicans* transcripts of more than 500-fold and in RNA-seq libraries containing more than 50% of fungal transcripts. To verify that neither this enrichment nor the predominating background of host material introduced a bias in the results, a simple validation was carried out. RNA from *C. albicans* was mixed with host RNA (1% of *C. albicans*, 99% of uninfected host). This spiked RNA was next subjected to enrichment with the biotinylated bait system. The results obtained from sequencing these samples were compared to those of sequencing the same *C. albicans* RNA without the presence of host RNA and without enrichment. Using this approach, we were able to verify that there was no bias for 97% of the genes. Therefore, the relative amount of each gene was the same whether the samples were subjected to the enrichment procedures or not. Moreover, a machine learning approach helped to delimitate the necessary features for the baits in order to efficiently capture their target genes. Such information will be useful for future designs of bait libraries, either targeting *C. albicans* or other organisms.

This enrichment method allowed RNA-sequencing of *C. albicans* from infected mouse kidneys, after 16 and 48 h of infection, and from infected *Galleria mellonella* larvae, 2 and 24 h post-infection. These samples revealed a high resolution transcriptome, showing the expression levels of over 80% of all *C. albicans* genes, constituting a huge improvement in resolution relatively to previous *in vivo* transcriptional analyses of this microorganism. Over 1000 genes were found to be statistically up- or down-regulated *in vivo* relatively to *in vitro* when performing a meta-analysis that identified genes commonly regulated in the four different conditions. Several functions were enriched among these genes, including some typically associated with virulence, such as adhesion, iron homeostasis, stress response, response to starvation, and biofilm formation. On the other hand, such detailed landscape of the *C. albicans* gene expression profile allowed the identification of a large number of genes that were so far ignored to participate in the process of host invasion and infection, and these alone will be targets of investigation for years to come.

The two models of infection, mouse and insect larvae, elicited surprisingly similar transcriptional responses from *C. albicans*, highlighting the adequation of this insect model to study *C. albicans* virulence. These data are consistent with studies that revealed a good correlation between *C. albicans* virulence in mice invasive models and the insect larvae (Brennan et al., 2002; Amorim-Vaz et al., 2015a). Unfortunately, the libraries enriched for *C. albicans* cannot be used to analyze the host transcriptome without a non-negligible bias. Still, dual RNA-seq of host and pathogen can be performed on the same RNA sample, if both a non-enriched and an enriched library are prepared and sequenced.

**FIGURE 3:**
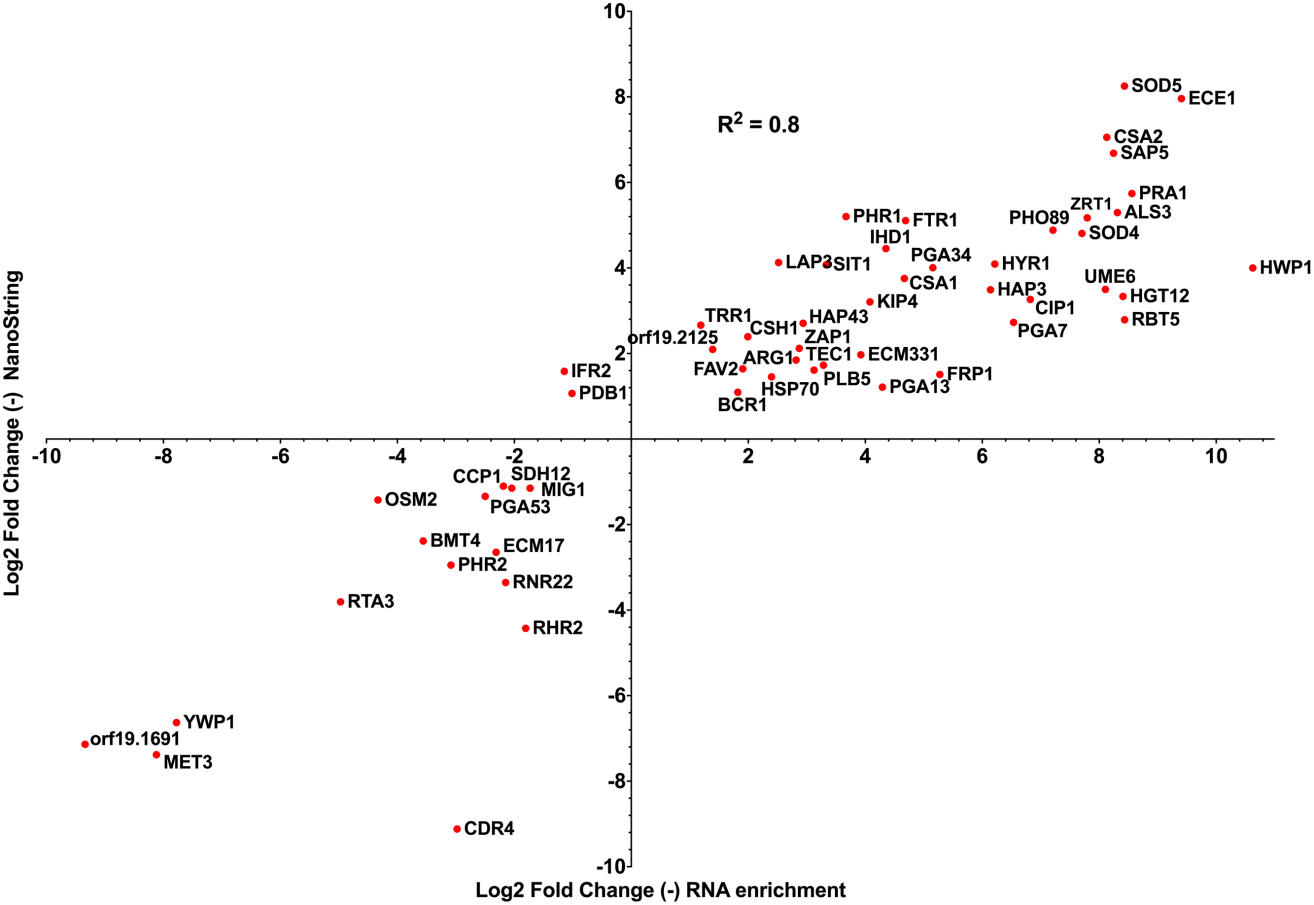
**Correlations between log2 fold change data generated by nanoString and enrichment/RNA-seq approaches (Amorim-Vaz et al., 2015b; Xu et al., 2015), taking early gene expression patterns from both studies (nanoString: mouse kidneys 12 h post infection versus *in vitro* stationary-phase culture; enrichment/RNA-seq: mouse kidneys 16 h post infection versus *in vitro* exponential-phase culture).** The total number of genes regulated at early time points in Xu et al. (2015) and Amorim-Vaz et al. (2015b) was 148 and 1114, respectively. *R*^2^: *R* squared Pearson correlation coefficient, calculated with Prism 6.0.

Taking datasets produced from both technologies analyzing *C. albicans* systemic infection in mice, we compared sets of *C. albicans* genes that were common between both studies and estimated the expression levels as compared to *in vitro* grown cells. We considered only early infection times of both studies for the comparison and only genes that were significantly regulated as compared to *in vitro* conditions. With a set of 56 genes in common between both studies, the data showed a high correlation (*R*^2^: 0.8) between observed gene expression levels (**Figure 3**). This high correlation in gene expression profile is remarkable, given the different experimental conditions between both studies (choice of time points, choice of mice strain) and the different analytical approaches taken. Among the genes that were commonly upregulated in both data sets, genes relevant for *C. albicans* pathogenesis can be identified including: (i) *HWP1* (hyphal specific protein involved in adhesion to epithelial cells) and *ALS3* (adhesin involved in the adherence of *C. albicans* to endo- and epithelial cells), (ii) *ZRT1* and *PRA1* (zinc transporter and zinc binding protein important for zinc acquisition of *C. albicans*) and (iii) *FTR1* (a high-affinity iron permease) and *SIT1* (a transporter of ferrichrome siderophores). These results suggest that the enrichment/RNA-seq method can be an alternative to nanoString when whole genome data are required.

## Conclusion and Perspectives

The two novel transcriptional approaches, nanoString and RNA-seq following enrichment, represent powerful tools to analyze the behavior of pathogens during infection. With RNA samples directly taken from within its host, the technical challenges of quantification of fungal mRNA in a complex host mixture have now been overcome, with the difference that nanoString targets still a limited number of genes of interest while enrichment/RNA-seq is genome-wide. Future applications of enrichment/RNA-seq may include the transcriptome analysis of *C. albicans* mutants with reduced virulence. So far, this task was even more challenging than for wild-type strains due to the reduced fungal burdens often found for such mutants in host organs, thus further reducing the proportion of fungal versus host biomass. With the potential of these transcriptome technologies, it will be also possible to investigate *C. albicans* gene expression in other contexts, such as from different organs during systemic infection or during other types of infections, like oropharyngeal candidiasis, mucosal infections or gastrointestinal colonization. Importantly, since these methods allow the selective sequencing of fungal transcripts, it will be now possible to perform such studies directly on human biopsies from solid tissues, even if containing low amounts of fungal cells. Such data will be useful to estimate to which extent the *C. albicans* transcriptome from clinical samples differs from animal models. Moreover, the possible applications of transcriptome technologies are obviously not limited to *C. albicans*. To apply them to other pathogens, one needs simply to design a set of probes matching the ORFome of the organism of interest. Both approaches currently implicate significant costs given their technical specificities and the need to perform experiments in duplicates or triplicates (for example about 600 USD per sample for analysis of 500 genes by nanoString; about 500 USD for SureSelect application and RNA-seq per sample for whole *C. albicans* genome). The significant costs of these technologies are originating from capture probe biosynthesis, which might be reduced in the future by novel chemistry. On the other hand, the expansion of their future use will probably contribute to decrease their price.

## Conflict of Interest Statement

The authors declare that the research was conducted in the absence of any commercial or financial relationships that could be construed as a potential conflict of interest.
